# IDO and intra‐tumoral neutrophils were independent prognostic factors for overall survival for hepatocellular carcinoma

**DOI:** 10.1002/jcla.22872

**Published:** 2019-03-06

**Authors:** Yan Wang, Rongrong Yao, Lan Zhang, Xiaoying Xie, Rongxin Chen, Zhenggang Ren

**Affiliations:** ^1^ Key Laboratory of Carcinogenesis and Cancer Invasion Ministry of Education China; ^2^ Liver Cancer Institute, Zhongshan Hospital Fudan University Shanghai China; ^3^ Department of Oncology, Huashan Hospital Fudan University Shanghai China

**Keywords:** HCC, IDO, neutrophils

## Abstract

**Background:**

Both Indole‐amine‐2,3‐dioxygenase (IDO) and neutrophils were proved to have pro‐tumor effect in some kinds of solid tumors by immune suppression. However, little is known about their effect on hepatocellular carcinoma (HCC) and the relationship between these two immune‐suppressive factors. The aim of this study was to evaluate the prognostic significance of IDO and intra‐tumoral neutrophils and their correlations in HCC.

**Methods:**

Specimens’ tissue microarray (TMA) for 153 HCC patients was used in this study. We examined intra‐tumoral expression of IDO and CD66b in TMA. The Kaplan‐Meier method and Cox regression models were used to evaluate the prognostic value of IDO expression and CD66b.

**Results:**

Multivariate analysis showed both IDO expression and intra‐tumoral neutrophils infiltration were independent prognostic factors for overall survival (OS). In high IDO expression group, the percentage of intra‐tumoral neutrophils infiltration was higher than that in low IDO expression group.

**Conclusion:**

Both IDO and intra‐tumoral neutrophils were independent prognostic factors for overall survival for HCC.

## 1 INTRODUCTION

Hepatocellular carcinoma (HCC) is a leading cause of cancer‐related death worldwide.^1^ Although a growing number of molecular‐targeted drugs and immune therapy proved can be of benefit in part of patients with advanced HCC, the high price and low objective response rate make majority of patients cannot benefit from them.^2^ It is of great importance to seek optimal biomarkers to improve the outcome of advanced HCC.

Indole‐amine‐2,3‐dioxygenase (IDO) is responsible for the first enzymatic step of tryptophan catabolism by the kynurenine pathway performed as the rate‐limiting enzyme. Protein expression of IDO was found to be high in a number of tumor samples and contribute to decrease patient survival.^3^, ^4^ IDO expression in various histologic cancer types seems to build an immune‐suppressive microenvironment^5^, ^6^ by regulating immune cells such as T effector cells,^7^ Treg cells,^8^ and MDSC.^9^, ^10^ On the other hand, several studies have provided compelling evidence for pro‐tumor functions of neutrophils by immune suppression.^11^, ^12^ However, little was known about the relationship between IDO and neutrophils while tumor‐associated neutrophils (TANs) were found in various human cancers.^13^, ^14^

In this study, we investigated the prognostic significance of IDO and intra‐tumoral neutrophils in HCC patients and explored the correlation between IDO and intra‐tumoral neutrophils.

## 2 METHODS

### 2.1 Study population

HCC specimens used in tissue microarray (TMA) were collected from patients who underwent radical resection from January 2008 to December 2008 at Liver Cancer Institute, Zhongshan Hospital (Fudan University, Shanghai) with informed consent form signed off. A total of 153 patients who had not received anticancer therapy before surgery and there was no sign of distant metastasis were enrolled in the study. Follow‐up tests included ultrasound, AFP measurements (every 2‐3 months), and contrast‐enhanced CT or MRI (every 6 months). The last follow‐up was in April 2016. The study was approved by the research ethics committee of Zhongshan Hospital.

### 2.2 TMA and immunohistochemical staining

Two cylinders of the tumor tissue were included in each case in TMA to ensure reproducibility and homogeneity. Intra‐tumoral neutrophils were evaluated by immunohistochemical staining of CD66b, which was a marker mainly expressed by human neutrophils.^15^, ^16^ A two‐step method of immunohistochemistry (IHC) including a heat‐induced antigen‐retrieval procedure was performed. The primary antibodies used were anti‐CD66b antibody (1:50, abcam) and anti‐IDO antibody (1:150, abcam). Microarrays were evaluated at 400× magnification light microscopy by pathologists blinded to the clinic pathologic data of the patients. IDO staining was evaluated by a score calculated by multiplying the staining extent score (0: 0%‐5%, 1: 6%‐25%, 2: 25%‐50%, 3: >50%) with the staining intensity score (0: no staining, 1: weak, 2: strong, 3: very strong), resulting in a low (0‐4) expression level or a high (>4) level for each case by mean value of two spots. CD66b staining was determined as negative or positive.

### 2.3 Statistical analysis

Analysis was performed with SPSS 19.0 for Windows (SPSS, Chicago, IL). All consecutive data were expressed as mean ± standard deviation. Correlations between immunostaining parameters and clinic pathologic features were analyzed by *χ* ^2^ test and Fisher's exact probability test as appropriate. Univariate and multivariate analysis were carried out with the Kaplan‐Meier method and the Cox proportional hazards regression model and was compared with the log‐rank test*.* *P* < 0.05 was considered statistically significant.

## 3 RESULTS

### 3.1 Prognostic factors for HCC patients after resection

**Figure 1:**
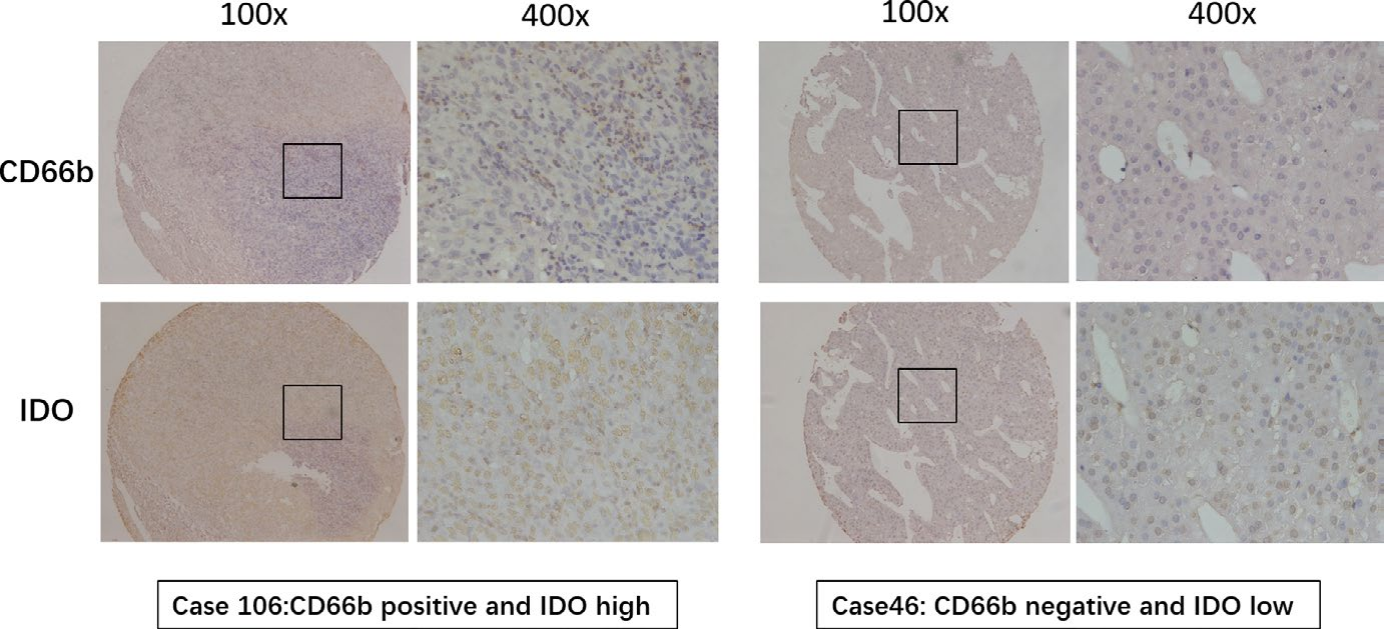
Representative images of IDO and CD66b staining

**Figure 2:**
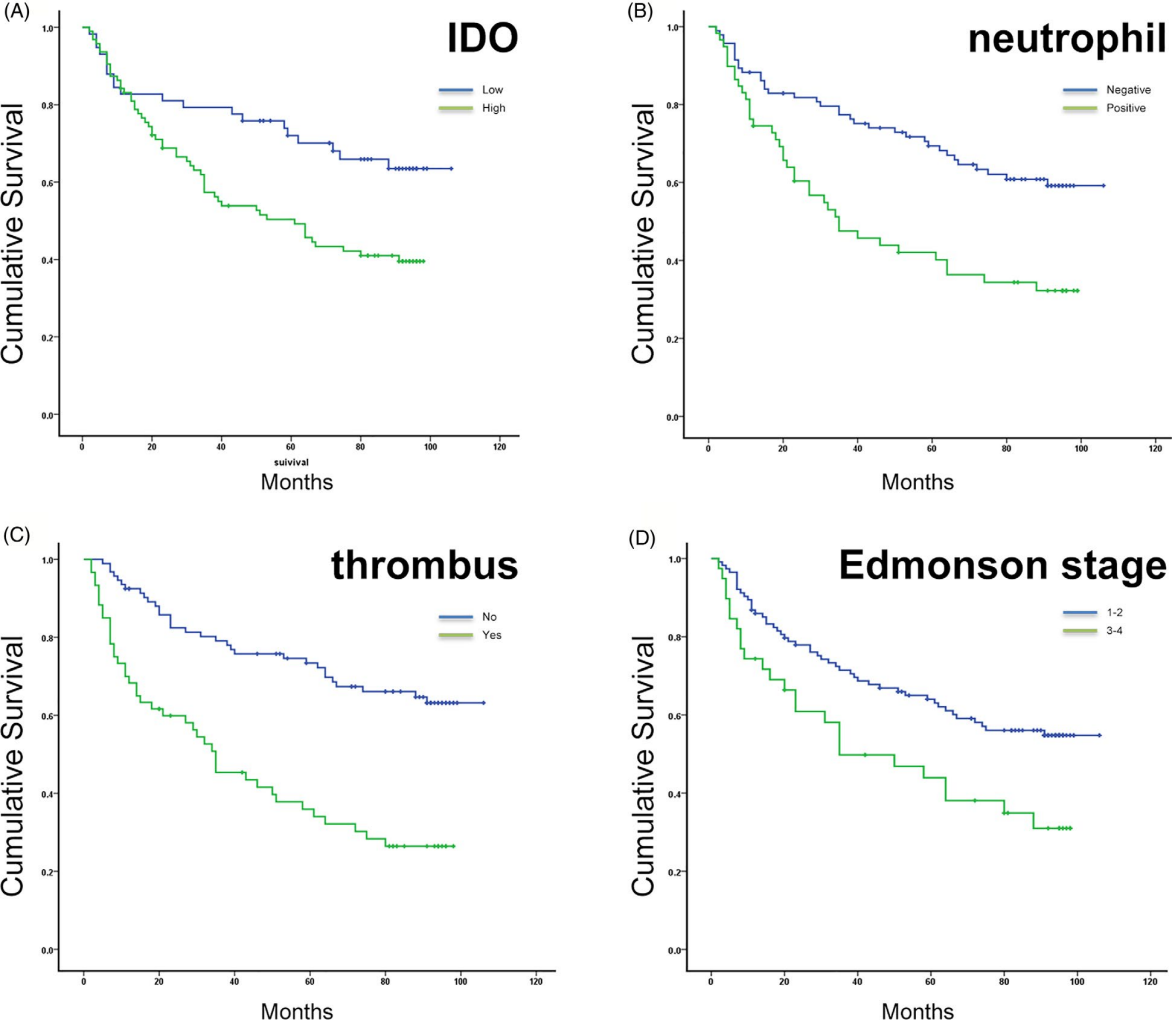
Survival curves of different groups. A, survival curves of high IDO expression group and low IDO expression group (OS 57.03 vs 79.4 mo, *P* = 0.007). B, survival curves of intra‐tumoral neutrophil positive group and negative group (OS 50.15 vs 77.28 mo, *P* = 0.000). C, survival curves of with thrombus (portal vein) group and without thrombus group (OS 45.33 vs 80.67 mo, *P* = 0.000). D, survival curves of Edmonson stage low (1‐2) group and high (3‐4) group (OS 74.54 vs 51.03 mo, *P* = 0.008)

The 1‐, 3‐, 5‐, 7‐years survival rates for 153 HCC patients after resection were 84%, 66%, 59% and 50%, respectively. Representative images of IDO and CD66b were shown in Figure 1. Univariate analysis showed that serum AFP level (more than 20 ng/mL) before resection, tumor diameter (more than 10 cm),with thrombus (portal vein), high Edmonson stage (stage 3‐4), high IDO expression, and intro‐tumoral neutrophils infiltration were prognostic factors for OS after resection (Table 1).Multivariate analysis showed high IDO expression (HR = 1.793, *P* = 0.028), intra‐tumoral neutrophils infiltration (HR = 2.159, *P* = 0.001), with thrombus (HR = 2.526, *P* = 0.000), and high Edmonson stage (HR = 1.848, *P* = 0.014) were independent prognostic factors for OS (Figure 2).

**Table 1:** Univariate and multivariate analyses of prognostic factors

	N	OS (mo)	Univariate	Multivariate	
			*P* value	HR (95% CI)	*P* value
Age					
>65 y	26	58.95	0.408		NA
<=65 y	127	68.96			
Gender					
Male	129	66.18	0.108		NA
Female	24	57.42			
Total bilirubin (µmol/L)					
<=17.1	126	69.19	0.430		NA
>17.1	27	57.83			
Albumin (g/L)					
>=35	142	68.81	0.341		NA
<35	11	49.87			
Prothrombin time (s)					
<=14	138	68.20	0.840		NA
>14	15	59.87			
AFP (ng/mL)					
<=20	59	72.83	0.031*	1.252 (0.747‐2.099)	0.394
>20	94	61.86			
HBsAg					
Positive	132	67.74	0.866		NA
Negative	21	63.43			
Cirrhosis					
No	26	64.77	0.744		NA
Yes	127	67.38			
Tumor diameter (cm)					
<=10	128	72.84	0.001*	1.409 (0.777‐2.557)	0.259
>10	25	39.25			
Tumor number					
Single	130	67.57	0.188		NA
Multiple	23	54.85			
Tumor capsule					
Yes	74	67.49	0.262		NA
No	79	64.37			
Thrombus					
No	93	80.67	0.000*	2.526 (1.509‐4.228)	0.000*
Yes	60	45.33			
Edmonson stage					
1‐2	114	74.54	0.008*	1.848 (1.132‐3.016)	0.014*
3‐4	39	51.03			
IDO					
Low	58	79.49	0.007*	1.793 (1.065‐3.019)	0.028*
High	95	57.03			
Neutrophil					
Negative	94	77.28	0.000*	2.159 (1.355‐3.440)	0.001*
Positive	59	50.15			

* *P < *0.05.

### 3.2 Correlation between IDO and intra‐tumoral neutrophils

To further understand the effect of IDO for HCC and its’ correlation with intra‐tumoral neutrophils, we compared clinical characteristics and percentages of patients with intra‐tumoral neutrophils infiltration between high IDO expression group and low IDO expression group. There was no difference in clinical characteristics between two groups (Table 2). However, it showed significant difference in percentages of patients with intro‐tumoral neutrophil infiltration between these two groups. In high IDO expression group, the percentage of intra‐tumoral neutrophils infiltration was 45.26%, much higher than that in low IDO expression group (27.59%, *P* = 0.022, Table 2), OR value was 2.171, and 95% CI was 1.074‐4.386. It confirmed that high IDO expression was a risk factor for intra‐tumoral neutrophils infiltration in HCC patients.

**Table 2:** Difference between IDO high group and IDO low group of clinical characteristics and intra‐tumoral neutrophils

	IDO high group n = 95	IDO low group n = 58	*P* value
Age			
>65 y	14	12	0.379
<=65 y	81	46	
Gender			
Male	80	49	1.000
Female	15	9	
Total bilirubin (µmol/L)			
<=17.1	79	47	0.828
>17.1	16	11	
Albumin (g/L)			
>=35	87	55	0.535
<35	8	3	
Prothrombin time (s)			
<=14	86	52	1.000
>14	9	6	
AFP (ng/mL)			
<=20	31	28	0.061
>20	64	30	
HBsAg			
Positive	81	51	0.810
Negative	14	7	
Cirrhosis			
No	16	10	1.000
Yes	79	48	
Tumor diameter (cm)			
<=10	79	49	1.000
>10	16	9	
Tumor number			
Single	81	49	1.000
Multiple	14	9	
Tumor capsule			
Yes	48	31	0.742
No	47	27	
Thrombus			
No	55	38	0.396
Yes	40	20	
Edmonson stage			
1‐2	69	45	0.569
3‐4	26	13	
Neutrophil			
Negative	52	42	0.022*
Positive	43 (45.26%)	16 (27.59%)	

* *P < *0.05.

## 4 DISCUSSION

The results of this study showed that both high IDO expression and intra‐tumoral neutrophils infiltration were independent prognostic factors for poor survival for HCC patients. There are three Trp‐catabolic enzymes (IDO1, IDO2, and TDO) in mammals which catalyze conversion of the essential amino acid tryptophan (Trp) to kynurenine (Kyn). In humans, IDO1 shows a high protein expression in the peripheral lymph organs, while IDO2 and TDO show high tissue specificity and much lower expression level than IDO1 that significantly restrict their activity.^6^, ^17^ The “IDO” we discussed in this study was IDO1. In patients with solid tumors, such as colorectal cancer, small cell lung cancer, melanoma, and ovarian cancer, high IDO expression is correlated with a poor prognosis and shorter overall survival.^18^, ^19^ In HCC, IDO was expressed in HCC cells following the stimulation of IFN‐γ,^20^ and our study confirmed that high IDO expression was a prognostic factor for poor survival for HCC patients. IDO modifies inflammation and immunity through a variety of effector cells: induces the differentiation of Treg cells and apoptosis of effector T cells,^7^ prevents Treg cells destabilization and maintains the suppressive phenotype,^8^, ^21^ recruits and activates MDSCs to suppress antitumor immune responses,^9^, ^10^ inhibits the surface expression of activating receptors and regulates NK‐cell function.^22^, ^23^ There is strong evidence that suppression of antitumor immune responses by IDO would make such catabolism an attractive target for therapeutic intervention.^24^

Clinical evidence indicates that neutrophils are involved in tumor progression. A negative correlation between the number of tumor‐associated neutrophils and prognosis has been evidenced for many types of cancer including renal carcinoma,^25^ colorectal cancer,^26^ gastric cancer,^27^ HCC,^28^ and non‐small cell lung cancer.^29^ Our results showed that intra‐tumoral neutrophils infiltration was an independent prognostic factor for poor survival for HCC patients, which is consistent with previous results. Furthermore, we found that the percentage of intra‐tumoral neutrophils infiltration was much higher in high IDO expression group than that in low IDO expression group. This result indicated that IDO might play a role in recruitment of neutrophils. Like other immune cells, TANs emerge in tumors by recruitment from the blood. CXCR2 up‐regulated by IL‐8 or IFN‐β promote neutrophils recruitment.^30^, ^31^ CXCL5 promote intra‐tumoral neutrophil infiltration through PI3K‐Akt and ERK1/2 signaling pathways.^32^ However, no direct evidence shows IDO can promote neutrophils recruitment in HCC. Future research and data should be provided regarding to the relationship between IDO and neutrophils.
